# Ironing Out the Deficiency: Tracking Iron in Celiac Disease Before and After the Gluten-Free Diet

**DOI:** 10.3390/nu18040590

**Published:** 2026-02-11

**Authors:** Patricia Dillawn, Sadie Nagle, Edwin Liu, Marisa Gallant Stahl

**Affiliations:** 1 Department of Pediatrics, University of Colorado School of Medicine, Aurora, CO 80045, USA; patricia.dillawn@childrenscolorado.org (P.D.);; 2 Department of Clinical Nutrition, University of Colorado School of Medicine, Aurora, CO 80045, USA; sadie.nagle@childrenscolorado.org; 3 Colorado Center for Celiac Disease, Digestive Health Institute, Children’s Hospital Colorado, Aurora, CO 80045, USA

**Keywords:** celiac disease, iron deficiency, iron deficiency anemia, gluten-free diet, iron supplementation

## Abstract

Celiac disease (CeD) is a gluten-induced immune-mediated enteropathy that preferentially involves the proximal duodenum. Consequently, iron deficiency is common in CeD, impacting at least 10% of newly diagnosed individuals. In this narrative review, we aim to investigate the mechanisms, prevalence, treatment, and monitoring of iron deficiency and the impacts of a gluten-free diet (GFD) on iron deficiency in individuals with CeD. We will also review the role of and approach to iron supplementation in this population. Specifically, we will explore whether mucosal healing on a GFD is sufficient for the management of iron deficiency amongst individuals with CeD.

## 1. Introduction

Iron deficiency with or without anemia frequently occurs as an extraintestinal manifestation of celiac disease (CeD). Gluten-induced enteropathy primarily affects the proximal duodenum, which is the main site for heme and non-heme iron absorption, thus increasing the risk of impaired iron uptake [1]. While strict adherence to a gluten-free diet (GFD) remains the cornerstone of CeD treatment and promotes mucosal healing, normalization of the iron status is not consistently achieved. Multiple cohort studies and nutritional analyses have documented persistent or recurrent iron deficiency with and without anemia in patients who maintain a strict GFD, even when structured follow-up and dietetic counseling are provided [2,3].

This review aims to explore the pathogenesis and epidemiology of iron deficiency in patients with CeD and summarize the evidence on the role of iron supplementation in treatment to help inform care. The objectives are to assess the adequacy of mucosal recovery on GFD alone for correcting iron deficiency, to compare outcomes between GFD-only and GFD-plus-iron-supplementation strategies, and to identify key areas for future research. This review will also provide our suggested approach to the treatment of children with CeD and iron deficiency, as defined by age-specific ferritin treatment level thresholds.

## 2. Methods

A literature search on the computer database PubMed used the following MeSH terms: “iron deficiency” and “celiac disease”. Articles from January 2013 to December 2025 were considered for inclusion. Only articles in the English version were included. This search strategy identified a total of 280 studies, of which 43 studies were included. An additional 16 studies were obtained through reviewing the references of the studies selected from the literature review. An additional 7 articles were reviewed for assessing different iron supplementation formulations. Full text and clinically relevant articles were used for this review.

## 3. Pathophysiology of Iron Absorption and Deficiency in CeD

Dietary iron comes in two forms: heme iron, found in meats, poultry, and fish, which is largely bioavailable, and non-heme iron, found in vegetables, fortified foods, and supplements, and which has a lower absorption rate that first requires the iron to be reduced from ferric (Fe^3+^) to ferrous (Fe^2+^) forms. This reduction in non-heme iron in the duodenum is enhanced by gastric acid and vitamin C. A typical Western diet contains up to 20 mg of daily iron, of which up to 10% is absorbed. Most of this is in the non-heme form that requires reduction to Fe^2+^ before absorption [4]. The primary site of iron absorption is in the duodenum and proximal jejunum, which is also the area affected by active CeD. Once iron is absorbed by the enterocyte, it is stored as ferritin or exported into the circulation by ferroportin. Ferroportin is the only known cellular iron exporter in humans. It transports iron into the bloodstream, and is found on enterocytes, macrophages, hepatocytes, and the placenta. On the other hand, hepcidin, made by the liver, functions to block ferroportin in order to control systemic iron availability and regulate iron homeostasis. Therefore, there is a balance between ferroportin making iron bioavailable from stores and hepcidin decreasing iron release from cells including enterocytes, which results in fecal losses of iron [5]. However, once iron is inside the enterocyte, most is absorbed across the basolateral membrane and then oxidized to Fe^3+^ by hephestin, then bound by transferrin in the plasma. Iron bound to transferrin enters the circulation, where it is delivered to sites for use and storage. Hepcidin increases in states of inflammation and infection, forming the basis of anemia of chronic disease, which also contributes to iron deficiency in CeD.

In CeD, when enterocytes of susceptible individuals are exposed to gluten peptides, an autoimmune response is triggered, leading to villous atrophy and, as a result, a decreased surface area on which to absorb iron. Accordingly, iron deficiency in CeD is thought to be primarily related to the mutual sites of iron absorption and villous atrophy in the duodenum, leading to malabsorption of iron. In children with active CeD, there is increased expression of ferroportin and decreased expression of hephestin compared to controls without CeD. However, this study failed to show a link to iron deficiency anemia, making the mechanistic interpretation unclear [6]. There may be other mechanisms involved, however, that may also explain the heterogeneity of iron deficiency in CeD. Some studies suggest that elevations in hepcidin in the state of small intestinal inflammation may also contribute to iron deficiency in CeD through iron sequestration [7]. The divalent metal transporter (DMT1) on enterocytes helps iron to enter the cell and upregulates in CeD despite the presence of villous atrophy, and a variant of DMT1 is associated with a higher risk of anemia that may be unmasked in CeD in those affected [8]. On the other hand, HFE variants (associated with hemochromatosis) can even play a protective role in iron deficiency anemia [9].

Genetic predisposition to lower iron levels appears to be causally related to a higher risk for CeD, as suggested by four iron-related SNPs. This supports the possibility that iron deficiency might not only be a consequence of CeD but may also predispose someone to its development [10]. The variation in iron status in CeD patients and their response to the GFD is unpredictable, and there may be underlying genetic factors that contribute to the rate and ability to recover iron stores in patients with CeD and iron deficiency.

Other mechanisms of iron deficiency to consider in CeD include occult gastrointestinal bleeding, which can occur from intestinal lesions due to intestinal gluten-mediated injury or from chronic gastritis, which is commonly associated with CeD [11], and gastrointestinal infection (e.g., H. Pylori).

## 4. Presentation and Prevalence

Symptoms of iron deficiency are clinically heterogeneous. Classically, patients often demonstrate physical fatigue (83%), cold intolerance (13%), and pica (8%) [12]. Other symptoms include neurologic symptoms, such as headaches, poor memory, poor concentration, visual disturbances, and depression or anxiety, cardiac symptoms, such as chest pain, shortness of breath, and palpitations, and integumentary symptoms, such as dry skin and brittle hair and nails [12]. Iron deficiency with or without anemia is common in individuals with CeD and CeD is also more common among those with iron deficiency anemia. In a meta-analysis of 18 studies, Mahadev et al. demonstrated a prevalence of biopsy-confirmed CeD in individuals with iron deficiency anemia of 3.8%, which is higher than the general population [13]. While there was significant heterogeneity among studies, the reported prevalence did not vary when adjusted for region and sex [13]. Therefore, while other biologic and dietary factors may contribute to iron deficiency and/or anemia, CeD is also an independent contributing factor [14]. Several studies also show that iron deficiency with or without anemia is common at the time of CeD diagnosis and follow-up both in the adult and pediatric population [15,16,17,18,19,20,21]. It follows that several society guidelines, including the North American Society of Pediatric Gastroenterology, Hepatology, and Nutrition (NASPGHAN), the European Society of Paediatric Gastroenterology, Hepatology, and Nutrition (ESPGHAN), and the American College of Gastroenterology (ACG), recommend iron deficiency and anemia screening in CeD patients [22,23,24]. In one single-center registry study of 458 children with CeD, 12% had anemia and 29% had iron deficiency based on ferritin levels [25]. Interestingly, while iron deficiency and/or anemia were the most common abnormalities at diagnosis in this study, iron deficiency anemia screening was also the most expensive component of the diagnostic laboratory evaluation. Since all cases of iron deficiency and anemia were captured with a ferritin level and complete blood count, costs could be minimized by eliminating an iron panel in the initial evaluation [25].

With an increased prevalence of iron deficiency with or without anemia in individuals with CeD, providers should have a low threshold to screen for CeD in the assessment of individuals with iron deficiency with or without anemia, although studies show that this overwhelmingly has not been implemented in clinical practice [26,27,28]. Amongst gastroenterologists, one study demonstrated that duodenal biopsies for CeD are cost effective and should be considered in adults undergoing endoscopy for iron deficiency anemia regardless of CeD serology status [29].

Some studies [17,21,30,31], but not all [19], have shown that iron deficiency with or without anemia corresponds with the degree of enteropathy, with higher Marsh scores corresponding with an increased prevalence of iron deficiency and anemia. This finding is supported by the proposed pathogenesis of iron deficiency in CeD with impaired iron absorption in the proximal duodenum. However, chronic inflammation also likely contributes to pathogenesis with mild elevations in hepcidin observed in individuals with CeD and iron deficiency [7]. Therefore, despite this correlation with enteropathy, iron deficiency with or without anemia often does not correspond with gastrointestinal symptoms [32] and can commonly be found even in those with screening-identified or potential CeD [17,30]. This once again supports a low threshold for CeD screening in those identified to have iron deficiency with or without anemia.

## 5. The Impact of Treatment with a GFD on Iron Status

Strict adherence to the GFD is essential to promote mucosal healing and subsequently restore the iron absorptive capacity. Both adult and pediatric studies have demonstrated that CeD patients following the GFD have significant improvement in associated anemia after 6–12 months of a GFD and in ferritin levels after 24 months of a GFD regardless of iron supplementation [4,33,34,35]. However, iron deficiency can persist despite the GFD, which may reflect poor mucosal healing particularly in adults or those with poor dietary intake of iron [36,37,38,39,40,41]. The CADER study by Fernández-Bañares et al. followed seventy-six adult patients who were strictly adherent to the GFD, and after 2 years of monitoring, the persistence of villous atrophy was high (53%) [41]. In a US study in 2010, the median time to achieve mucosal healing in adult patients after strict adherence to the GFD was 3 years [42]. Children may demonstrate more rapid and consistent mucosal healing on the GFD, even within 3 months in some studies [43,44]. Despite this, some pediatric studies do demonstrate a substantial risk of persistent iron deficiency with or without anemia despite long-term GFD adherence [38]. For instance, a Dutch retrospective study (n = 130) reported that, between 3 months and 10 years post-diagnosis, 33% of pediatric patients had persistent low serum iron (μmol/L) and 21.9% had low serum ferritin (μg/L) [2]. Similarly, Spanish children and adolescents on the GFD for over a year exhibited higher rates of iron and folate deficiency than matched controls, despite comparable anthropometric measures and bone mineral density, suggesting that biochemical recovery may lag and is strongly influenced by dietary composition [3]. Prospective Polish data further corroborate these findings: after one year of GFD, iron deficiency remained prevalent without iron supplementation, and overall diet quality and subsequent iron deficiency showed minimal improvement without targeted nutrition education [45]. Importantly, children are also more impacted by the long-term neurocognitive sequelae of iron deficiency [46].

Multiple factors can affect iron status even after mucosal healing. Inflammatory mechanisms, such as hepcidin-mediated iron sequestration, may persist following gluten removal [1]. Furthermore, the GFD is often characterized by lower micronutrient density, limited iron fortification of processed foods, and a scarcity of naturally iron-rich foods [47]. Evidence consistently demonstrates that strict GFD dietary adherence alone does not ensure adequate iron intake [48]. Indeed, dietary intake represents a modifiable factor impacting iron status [4]. Targeted dietetic counseling that emphasizes both heme and non-heme iron sources, co-ingestion of vitamin C, and avoidance of inhibitors such as tea, coffee, and calcium is essential [45]. Furthermore, fortification strategies and the development of enhanced gluten-free product formulations may help prevent recurrent and persistent iron deficiency and associated anemia [47].

## 6. Iron Supplementation as an Adjunct to the GFD

The strongest direct evidence supporting iron supplementation as an adjunct to the GFD in patients with CeD comes from a randomized controlled trial (RCT) involving women with CeD and iron deficiency without anemia who had followed a GFD for at least one year. Over a 12-week period, oral ferrous sulfate supplementation (105 mg/day) produced significantly greater increases in ferritin compared to a high-iron GFD (>20 mg/day) alone [49]. These findings indicate that dietary modification alone is inadequate for timely normalization of iron stores, even when the absorptive capacity is presumably restored. Specifically, the mean ferritin increase was +22 µg/L in the supplementation group versus +5 µg/L in the high-iron GFD group, emphasizing the clinical relevance of iron supplementation in adults with other biologic factors [49]. Other studies that provide comparison between GFD-only and GFD-plus-iron-supplementation strategies are limited to retrospective cohort and observational studies. (Table 1—Studies comparing the iron status of CeD patients who were treated with the GFD alone versus the GFD with iron supplementation).

**Figure 1:**
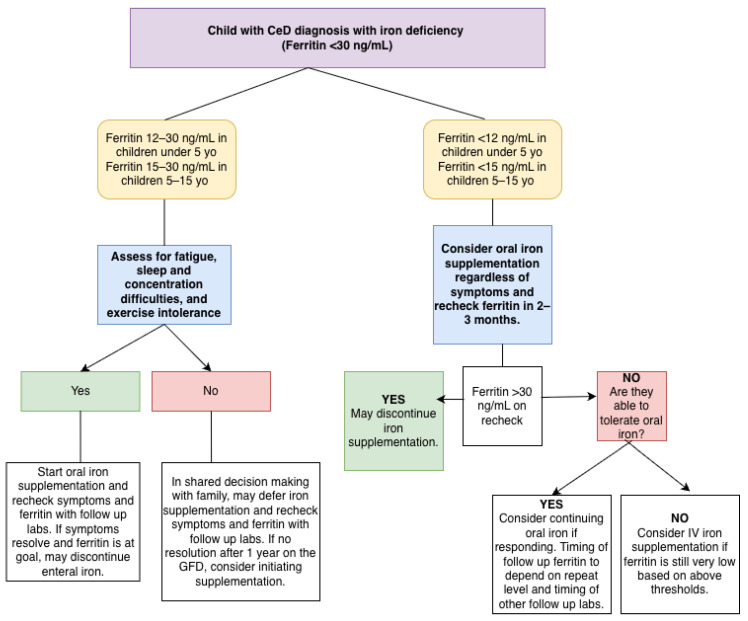
Approach to iron deficiency (ferritin < 30 ng/mL) in children with CeD.

There is no current consensus on when to provide iron supplementation as an adjunct to the GFD for CeD patients with iron deficiency (Figure 1—Approach to iron deficiency (ferritin < 30 ng/mL) in children with CeD). In Benson et al.’s expert consensus involving multiple subspecialties, including hematology and gastroenterology, treatment cut-offs of iron deficiency with or without anemia for all children less than 5 years old, children between 5 and 15 years old, and non-pregnant adults are defined as ferritin levels of <12 µg/L, <15 µg/L, and <50 µg/L, respectively [50]. Adult professional societies, such as the British Society of Gastroenterology (BSG) and American Gastroenterology Association (AGA), also recommend ferritin treatment cut-offs of <45–50 µg/L, with the provision that for adult patients with active inflammation, the treatment cut-off is <100 µg/L [51,52]. For pediatric CeD patients, the World Health Organization (WHO) maintains a similar provision as the BSG and AGA, providing a ferritin treatment cut-off of <30 µg/L for all children with evidence of chronic disease or active inflammation [53]. Additionally, pediatric professional societies, such as ESPGHAN, recommend iron supplementation for patients with iron deficiency anemia, as it is important to achieve the rapid recovery of iron stores to support childhood neurocognitive development [54]. Both ESPGHAN and NASPGHAN CeD monitoring guidelines do not maintain specific ferritin treatment thresholds [22,23].

Iron supplementation can be delivered orally or parenterally, and in practice is most often initiated orally. The AGA recommends starting with low-dose oral iron (administered daily or every other day with vitamin C) and switching to intravenous (IV) iron if oral therapy is poorly tolerated or ineffective, which is defined as ≤1 g/dL increase in hemoglobin in 4 weeks, or if malabsorption is suspected [52]. Pediatric guidelines also advocate for IV iron formulations in children who do not respond to or cannot tolerate oral iron, with additional recommendations such as monitoring for hypophosphatemia when using ferric carboxymaltose (Supplemental Table S1—Oral iron formulations; Supplemental Table S2—Intravenous iron formulations) [55]. It is important to consider iron supplementation particularly in the pediatric population and premenopausal adult female population, as they are at increased risk of iron deficiency due to increased physiological requirements and menstrual blood loss, respectively [2,45]. Overall, therapy should be continued until ferritin levels have normalized, and thus the total treatment duration is variable.

Persistent iron deficiency may be a sign of ongoing enteropathy in established celiac disease patients on a gluten-free diet. Repeat endoscopy and histologic evaluation can help determine whether iron deficiency despite supplementation is due to ongoing enteropathy which could warrant additional adjunctive therapies. Additionally, there is utility in investigating other causes of iron deficiency and anemia in these patients, including testing for other micronutrients, such as folate, B12, and vitamin D, evaluating for anemia of chronic disease by obtaining a full iron panel, and performing repeat EGD with biopsies to assess for additional sources of gastrointestinal blood loss [56].

## 7. Gaps in Evidence and Future Research Directions

Many observational studies documenting persistent iron deficiency with and without anemia lack concurrent duodenal histology, which limits the ability to differentiate between biochemical lag and ongoing malabsorption from mucosal injury. There is a need for multicenter prospective cohorts that integrate Marsh scores, dietary adherence measures, and serial iron biomarkers [1,2].

The inclusion of transferrin saturation, soluble transferrin receptor, reticulocyte hemoglobin (RET-He), and hepcidin in research protocols could clarify the mechanisms underlying treatment failure and inform earlier intervention strategies [1]. This could help guide the development of more targeted therapies and improve patient stratification for iron supplementation. Given the cost of these additional iron biomarkers, this should only be considered in clinical practice in the case of ongoing iron deficiency in patients who maintain strict GFD adherence and have been optimized on their iron supplementation. Pragmatic trials assessing structured high-iron GFD interventions, fortification of gluten-free products, and supportive food environments would provide evidence on how much diet-based measures alone can enhance iron outcomes, thereby informing clinical recommendations regarding the necessity and timing of supplementation versus dietary optimization [3,45]. Research protocols should strive to unify the objective measurements for which they assess iron deficiency (e.g., ferritin treatment cut-offs for children 0–5 years, children 5–15 years, and adults of <12 µg/L, <15 µg/L, and <50 µg/L, respectively) and GFD adherence (e.g., gluten immunogenic peptides, validated dietary assessments) based on the study population.

A significant limitation in the existing literature is the inadequate evaluation of whether a gluten-free diet (GFD) supplies sufficient iron to meet age-specific requirements. This gap restricts the ability to distinguish persistent iron deficiency resulting from inadequate dietary intake from that due to ongoing malabsorption. Age-specific recommended dietary allowances (RDAs) for iron differ considerably: approximately 7–10 mg/day for young children (depending on age and sex), 15 mg/day for adolescent females, 18 mg/day for premenopausal women, and 8 mg/day for adult males and postmenopausal women [57]. Few studies have systematically quantified dietary iron intake relative to these standards. Scricciolo et al. found that even a high-iron GFD (>20 mg/day) was less effective than supplementation in adult women, indicating possible residual malabsorption [49]. In contrast, other studies [2,3,48] reported persistent deficiency without assessing whether the dietary intake met age-specific requirements. Future research should employ validated dietary assessments benchmarked against age-specific RDAs and incorporate mucosal healing status to distinguish dietary inadequacy, which can be addressed through counseling and fortification, from persistent malabsorption that necessitates supplementation.

Future adaptive RCTs comparing GFD-only strategies with algorithm-based supplementation could help determine which subgroups derive the greatest benefit from early oral or intravenous iron [49,52]. To date, we were only able to identify one RCT comparing GFD-only and GFD-plus-iron-supplementation strategies, which was conducted on a small population size of premenstrual women and thus cannot be broadly extrapolated to all CeD patients. Adult and pediatric CeD registries should incorporate defined monitoring intervals and escalation thresholds to assess the real-world outcomes of GFD-only versus GFD-plus-supplementation approaches, utilizing modern causal inference methods. To facilitate these trials, specific ferritin thresholds should be established as criteria for early IV iron intervention that also take into consideration age- and gender-specific differences. Suggested cut-off points include ferritin levels below 15 µg/L based on the above referenced guidelines, which may support protocol development among collaborators [58].

## 8. Conclusions

Although mucosal healing on a strict GFD is necessary, it is often insufficient to fully resolve iron deficiency in CeD. Long-term studies consistently document persistent deficiency in a significant proportion of patients, and an RCT has demonstrated superior ferritin recovery with oral iron supplementation compared to high-iron dietary interventions alone. Current guidelines recommend structured monitoring, low-dose oral iron with vitamin C, and intravenous iron when indicated [2,49,52]. Future research should prioritize the correlation of histology and iron biomarkers in longitudinal studies and pragmatic interventions to identify which patients will achieve normalization with diet alone and which require early supplementation. This strategy will improve personalization and guidance in managing iron deficiency in CeD [59]. The primary challenge for the coming decade is to determine the most effective modifiable factors to close the iron gap in CeD and to establish which strategies best integrate dietary modifications and supplementation to ensure iron adequacy for all patients.

## Figures and Tables

**Table 1:** Summarization of studies comparing the iron status of CeD patients on the GFD with and without iron supplementation.

Study	Population	Study Design	GFD Alone Outcomes	GFD and Iron Supplementation Outcomes	Key Findings
Ben-Ami et al. [33] *	60 CeD children and iron deficiency without anemia	Retrospective cohort, 12-month follow-up	Ferritin increased from 8.9 ± 3.8 to 18.6 ± 9.5 ng/mL (*p* < 0.001)	Ferritin increased from 9.0 ± 4.7 to 25.2 ± 20.8 ng/mL (*p* < 0.001)	No significant difference between groups (*p* = 0.46); most children normalized ferritin within 12 months on GFD alone
Scricciolo et al. [49] *	22 women with CeD on GFD with iron deficiency without anemia	Randomized clinical trial, 12 weeks	Iron-rich diet (>20 mg/day): ferritin levels remained low	Ferrous sulfate (105 mg/day): ferritin increased from 8.5 to 34 ng/mL (*p* = 0.002)	Iron supplementation significantly more effective than dietary intervention alone; similar tolerability in both groups
Saukkonen et al. [36] *	163 adults with CeD	Observational study, 1-year follow-up	Most recovered from anemia within 1 year on strict GFD without additional iron supplementation	Not separately evaluated	6% still had IDA after 1 year of GFD, especially women; slower mucosal recovery was identified in anemic patients
Studies assessing the iron status of CeD patients on the GFD with iron supplementation provided as needed					
Roldan et al. [34] *	572 adults with newly diagnosed CeD; 25% had anemia at diagnosis	Prospective cohort, 2-year follow-up	All patients received appropriate supplementation when needed based on deficiencies	81% normalized Hb within 1 year, 89% within 2 years with GFD and appropriate supplementation when needed	Iron deficiency present in 78.8% of anemic patients; most normalized Hb levels with GFD and targeted supplementation
Kreutz et al. [2] *	130 children with celiac disease; single-center cohort	Retrospective chart review, 3 months to 10 years after initiation of GFD	All patients received appropriate supplementation when needed based on deficiencies	Iron deficiency in 33% of measurements; ferritin deficiency in 21.9% of measurements	High prevalence of iron and ferritin deficiency persists during long-term follow-up despite GFD
Stahl et al. [35]	42 children with celiac disease identified through mass screening	Prospective cohort study; part of the Autoimmunity Screening for Kids (ASK) study	All patients received appropriate supplementation when needed based on deficiencies	Iron deficiency was common at baseline (21 of 24 children; 87.5%) and normalized at follow-up in 52.3% (11 of 21 children); majority of families reported good to excellent adherence to the GFD	Iron deficiency in children who have good adherence to the GFD with appropriate supplementation will resolve in 1 year

* The studies above assessed for adherence to the GFD through clinical monitoring by registered dieticians and/or gastroenterologists that specialize in CeD. Celiac Disease, CeD; gluten-free diet, GFD; iron deficiency anemia, IDA.

## Data Availability

No new data were created or analyzed in this study. Data sharing is not applicable to this article.

## Supplementary Materials

The following supporting information can be downloaded at https://www.mdpi.com/article/10.3390/nu18040590/s1, Table S1: Oral Iron Formulations [60,61,62,63,64]; Table S2: Intravenous Iron Formulations [65,66].
