# Supplementary material for: Ironing Out the Deficiency: Tracking Iron in Celiac Disease Before and After the Gluten-Free Diet
Source: Nutrients. 2026 Feb 11;18(4):590. doi: 10.3390/nu18040590 (PMC12943008; doi:10.3390/nu18040590)
Supplement: Supplementary file 1 [file nutrients-18-00590-s001.zip › nutrients-4091912-supplementary.pdf]

Supplemental Tables

Table 1. Oral Iron Formulations

Iron Form	Oxidation State	Approximate Percentage of Elemental Iron	Typical Tablet Equivalence	Average Absorption (Percentage of Dose)	Tolerability	Commercial Price (30 tablets)
Ferrous sulfate	Fe^{2+}	20% (65 mg elemental iron per 325 mg tablet)	325 mg tablet = 65 mg elemental iron	10–15% on empty stomach; reduced to ~4–6% with food [60]	Most common side effects: constipation (12%), nausea (11%), diarrhea (8%); GI symptoms in 32–70% of patients [62]	$0.30–$4.50
Ferrous fumarate	Fe^{2+}	33% (106 mg elemental iron per 325 mg tablet)	325 mg tablet = 106 mg elemental iron	10–15% on empty stomach; similar to ferrous sulfate [60]	Like with ferrous sulfate, GI side effects common	$3.90
Ferrous gluconate	Fe^{2+}	12% (27-38 mg elemental iron per 240–325 mg tablet)	325 mg tablet = 38 mg elemental iron	10–15% on empty stomach; similar to ferrous sulfate [60]	May be better tolerated than ferrous sulfate at equivalent doses; fewer GI symptoms reported	$1.50–$3.30
Ferrous succinate	Fe^{2+}	Variable (typically ~35%)	Varies by formulation	Similar to other ferrous salts [61]	Potentially better tolerability than ferrous sulfate [63]	Not widely available in US
Ferrous bisglycinate	Fe^{2+}	Variable (typically 20–25 mg elemental iron per tablet)	Varies by formulation; ~25 mg per tablet common	Similar to ferrous salts [61]	Better tolerated than ferrous sulfate	$2.40
Iron polymaltose complex	Fe^{3+}	Variable (typically 50–100 mg elemental iron per dose)	Varies by formulation	Lower absorption than ferrous salts; does not require acidic environment [60]	Better GI tolerability; fewer side effects than ferrous salts [64]	Variable; not widely available in US
Polysaccharide-iron complex	Fe^{3+}	Variable (product name indicates elemental iron content)	150 mg elemental iron per capsule typical	Does not require acidic environment; absorption comparable to ferrous salts [60]	Better tolerated; can be taken with meals without significant absorption reduction [64]	$4.20

Note: https://online.lexi.com/ was utilized to obtain common dosing and pricing of medications.

Table 2. Intravenous iron formulations						
IV Iron Formulation	Iron-Carbohydrate Complex	Elemental Iron per Dose	Typical Dosing Strategy	Infusion Time	Hypersensitivity Risk	Typical Insurance Coverage (United States)
Iron sucrose	Iron sucrose complex	100–300 mg	200 mg × 5 doses over 14 days or 300 mg × 3 doses over 28 days	15–60 minutes per dose	Lowest risk among all formulations; anaphylaxis rate <0.5% [65,66]	Generally covered for CKD patients; may require prior authorization for other indications
Ferric gluconate	Sodium ferric gluconate complex	125 mg	125 mg × 8 doses given no more often than every other day	60 minutes per dose	Low to moderate risk; higher than iron sucrose but lower than iron dextran [65,66]	Generally covered for CKD patients on hemodialysis; may require prior authorization for other indications
Low-molecular-weight iron dextran	Low-molecular-weight iron dextran complex	Up to 1000 mg	Single 1000 mg dose	~60 minutes for total dose infusion	Moderate risk; significantly lower than high-molecular-weight iron dextran; anaphylaxis rate ~0.6% [65,66]	Generally well-covered; most cost-effective option
Ferric carboxymaltose	Ferric carboxymaltose complex	750–1000 mg	750 mg × 2 doses one week apart or single 1000 mg dose	15 minutes per dose	Low risk; anaphylaxis rate <0.5%; risk of severe hypophosphatemia (50-74% biochemical hypophosphatemia) [65,66]	Generally covered for IDA with oral iron intolerance/failure and non-dialysis CKD; covered for heart failure with iron deficiency (NYHA II/III)
Ferumoxytol	Ferumoxytol (superparamagnetic iron oxide with carbohydrate shell)	510–1020 mg	510 mg × 2 doses 3–8 days apart, or single 1020 mg dose	15–30 minutes per dose	Moderate to high risk; highest anaphylaxis reporting rate among modern formulations; severe hypotension in 1.9% [65,66]	Generally covered for IDA with oral iron intolerance/failure and CKD; may require prior authorization
Ferric derisomaltose	Ferric derisomaltose (iron isomaltoside) complex	Up to 1000 mg	Single 1000 mg dose	≥20 minutes	Low risk; anaphylaxis rate <0.5%; lower hypophosphatemia risk (~8%) compared to ferric carboxymaltose [65,66]	Generally covered for IDA with oral iron intolerance/failure and non-dialysis CKD; newer agent may have more restrictive coverage

Note: https://online.lexi.com/ was utilized to obtain common dosing, duration, and infusion time of medications.

Disclaimer/Publisher's Note: The statements, opinions and data contained in all publications are solely those of the individual author(s) and contributor(s) and not of MDPI and/or the editor(s). MDPI and/or the editor(s) disclaim responsibility for any injury to people or property resulting from any ideas, methods, instructions or products referred to in the content.
